# The effectiveness of levothyroxine starting doses after thyroidectomy is compromised by early body variations occurring after surgery

**DOI:** 10.1007/s12020-026-04610-x

**Published:** 2026-04-27

**Authors:** Pierpaolo Trimboli, Pietro Giacopelli, Martina Zandonà, Verdiana Caironi, Chiara Camponovo, Elena Gamarra, Lauro Gianola, Barbara Lucchini, Marco Centanni, Mario Rotondi, Camilla Virili, Petra Makovac

**Affiliations:** 1 Thyroid Unit, Clinic for Endocrinology and Diabetology, Ente Ospedaliero Cantonale, Bellinzona, Switzerland; 2 Faculty of Biomedical Sciences, Università Della Svizzera Italiana, Lugano, Switzerland; 3 Service of Surgery, Regional Hospital of Mendrisio, Ente Ospedaliero Cantonale, Mendrisio, Switzerland; 4 Department of Internal Medicine, Ente Ospedaliero Cantonale, Bellinzona, Switzerland; 5 Sapienza University of Rome, Department of Medical-Surgical Sciences and Biotechnologies, Latina, Italy; 6 Department of Internal Medicine and Therapeutics, University of Pavia, Pavia, Italy; 7 Istituti Clinici Scientifici Maugeri IRCCS, Unit of Internal Medicine and Endocrinology, Laboratory for Endocrine Disruptors, Pavia, Italy

**Keywords:** Thyroid, Levothyroxine, Dose, Thyroidectomy, Hypothyroidism

## Abstract

**Background:**

Dosing levothyroxine (LT4) in athyreotic patients after total thyroidectomy may appear straightforward to manage. However, robust evidence to guide therapy remains lacking. This study aimed to provide further information on the efficacy of fixed, weight-adjusted LT4 dosing initiated immediately after surgery.

**Materials:**

Consecutive patients were enrolled at a single institution applying a standardized postoperative protocol: all cases initiated LT4 therapy immediately postoperatively at dose of ~1.6 mcg/kg, undergo their thyroid function test after six weeks, and receive subsequent dose adjustments until the target is reached.

**Results:**

Forty-eight patients met the inclusion criteria. The median LT4 starting dose was 100 mcg per day and 1.56 mcg/kg of body weight per day. At the first follow-up, 16 (33.3%) patients were at target. Over follow-up, the overall median time-to-target was 4 months. The 32 patients who reached the target later included 15 patients showing a decreased LT4 requirement and 17 patients with an increased LT4 need. The correlation analysis between body weight and LT4 dose revealed significant negative association between LT4 dose and weight variation (R² 0.18). In the multiple linear regression body weight and comorbidities were positively associated with LT4 dose on target, while age showed a negative association.

**Conclusions:**

Early postoperative weight changes influence significantly LT4 dose requirements, and age and comorbidities provided additional predictive value. These findings highlight the need for early follow-up and LT4 titration in patients not at target after initial evaluation.

## Introduction

Hypothyroidism affects millions worldwide and represents the second most common non-communicable endocrine disorder after diabetes mellitus [1]. In most of the cases it is caused by autoimmune thyroiditis, followed by total thyroidectomy for various indications, and only a minority of patients present with rarer thyroid dysfunctions or congenital dysgenesis. Regardless of etiology, a part of the transient or subclinical forms, all hypothyroid patients require lifelong levothyroxine (LT4) replacement to restore thyroid hormonal homeostasis [2]. In autoimmune and rare forms of hypothyroidism, LT4 therapy is individualized according to residual thyroid function. In contrast, athyreotic patients after total thyroidectomy are usually prescribed a full replacement dose of LT4 [3]. At first glance, this setting may appear the most straightforward to manage; however, robust evidence to guide therapy remains lacking [3]. No randomized clinical trials have been conducted. Current guidelines recommend initiating LT4 at 1.6–1.8 mcg/kg of body weight, with adjustments based on patient-specific factors such as lean body mass (often unavailable in routine practice), age, and comorbidities [4, 5]. Conversely, observational studies suggest starting with a fixed LT4 dose followed by titrations over time [6–10]. Moreover, a recent meta-analysis found similarly limited efficacy across different dosing strategies (weight-based vs. fixed dose) [11]. The challenge is further compounded by variability in the bioavailability of different LT4 formulations—tablet, soft-gel, or liquid—depending on concurrent medications and comorbidities [3, 12]. As a result, the optimal LT4 starting dose after thyroidectomy remains undefined. Although individualized therapy is widely advocated, local protocols often prevail [11]. Consequently, the time to achieve euthyroidism—time to target (TtT)—after total thyroidectomy is unpredictable, leaving patients concerned about the actual effectiveness of LT4 therapy.

The present study was undertaken to provide further information on the efficacy of fixed, weight-adjusted LT4 dosing initiated immediately after total thyroidectomy. Consecutive patients were enrolled at a single institution applying a standardized postoperative protocol.

## Methods

### Study conduction

The present study was conducted according to the STROBE (STrengthening the Reporting of OBservational studies in Epidemiology) statement [13].

### Institutional setting

Our institution, a public health hospital in the Canton of Ticino, is the leading regional center for thyroid disorders and thyroid surgery. Surgical cases are routinely reviewed by a multidisciplinary thyroid team coordinated by the institutional Thyroid Unit. According to local guidelines, patients undergoing total thyroidectomy: (1) initiate LT4 therapy immediately postoperatively at a standard dose of ~1.6 mcg/kg (with the exception of patients requiring TSH suppression who use higher doses); (2) receive an LT4 formulation determined by insurance reimbursement policies (tablet is universally covered, whereas soft-gel formulation is reimbursed only under optional covers); (3) undergo their first thyroid function test at six weeks to verify achievement of TSH targets (i.e., within the reference range in non-cancer patients; below the upper limit of normal in thyroid cancer cases); and (4) receive subsequent dose adjustments with repeat testing every six weeks until the target is reached. Clinical data are available in the institutional database and can be extracted for research purposes.

### Case selection

The study included patients undergoing total thyroidectomy during the period between February 2021 to April 2025. The study design included a three-step selection procedure: (a) adults undergone total thyroidectomy for all causes were searched in the institutional database, and they were selected when signing the institutional consent for research; (b) cases were selected if were available data about LT4 starting dose, patient’s body weight and body mass index (BMI), thyroid tests over postoperative follow-up, LT4 therapy on target, comorbidities and other medications; (c) cases were excluded when diagnosed with thyroid cancers requiring LT4 therapy aimed at suppressing TSH levels, were using any type of thyroid hormones or any medications affecting thyroid function before total thyroidectomy, or did not sign the consent to be included in studies.

### Ethics

The study protocol was approved by the Ethics Committee of Tessin Canton (Switzerland).

### Laboratory measurement

The thyroid tests were measured with Elecsys® e601 platform (Roche Diagnostics). In this assay, TSH, free-T3 and free-T4 reference ranges are 0.30–4.20 mIU/L, 3.1–6.8 pmol/L, and 12–22 pmol/L, respectively. The sensitivity for TSH is 0.006 mIU/L.

### Statistical analysis

Continuous parameters were expressed as median and interquartile range (IQR). Data were compared using nonparametric statistical tests for paired or unpaired data as appropriate with Mann-Whitney or Wilcoxon test. Frequencies were compared with chi-square test. Pearson’s correlation coefficient to evaluate the linear association between two continuous variables. The statistical significance was set at p  <  0.05. Analyses and figures were performed with © 2023 MedCalc Software Ltd and GraphPad Prism version 7 (GraphPad software, CA, USA).

## Results

### Case study

Forty-eight patients met the study inclusion criteria. Their median age of the study series was 56 years. Eighty-seven percent of them were female. The median body weight was 67.11 kg, and the BMI was 25.3 kg/m2. The median LT4 starting dose was 100 mcg per day and 1.56 mcg/kg of body weight per day. Eighty-three percent of patients used soft-gel caps formulation of LT4 while the remaining ones were on tablet LT4. Table 1 details the main features of the case study.

**Table 1:** Characteristics of the study series

Age, yrs (median, IQR)	56, 43.25–67
Gender, female/male (n)	42/6
Weight, kg (median, IQR)	67.11, 56.61–78.87
BMI, kg/m2 (median, IQR)	25.3, 21.93–29.55
LT4 starting dose, mcg per day (median, IQR)	100, 88–125
LT4 starting dose, mcg/kg per day (median, IQR)	1.56, 1.49–1.65
LT4 formulation, tablet/soft-gel caps (n)	8/40
Users of medications recognized as interfering with LT4 (n)	14 (pantoprazole n = 11, esomeprazole n = 2, dexlansoprazole n = 1)
Diagnosed with diseases that have potential to impair LT4 (n)	11 (esophageal reflux disease n = 6, celiac disease n = 1, gastro- multiple food allergies n = 1, previous sleeve gastrectomy n = 1, previous gastric bypass n = 1, previous laparoscopic anterior rectal resection n = 1)

*IQR*, interquartile range

### Analysis of patients at their first follow-up

At the first follow-up from surgery, there were 16 (33.3%) patients who reached target TSH using a median daily LT4 dose of 1.55 mcg/kg (1.50–1.65); the remaining 32 (66.7%) did not reach target TSH taking a similar median daily LT4 dose of 1.56 mcg/kg (1.48–1.62) with no significantly different therapy between the two groups (p = 0.490). No significant difference was observed between patients on target and the others regarding the presence of comorbidities potentially impairing LT4 efficacy (2/16 and 9/32, respectively, p = 0.293) and interfering medications (6/16 and 13/32, respectively, p = 1.0).

### Data of all patients when on target

**Fig. 1:**
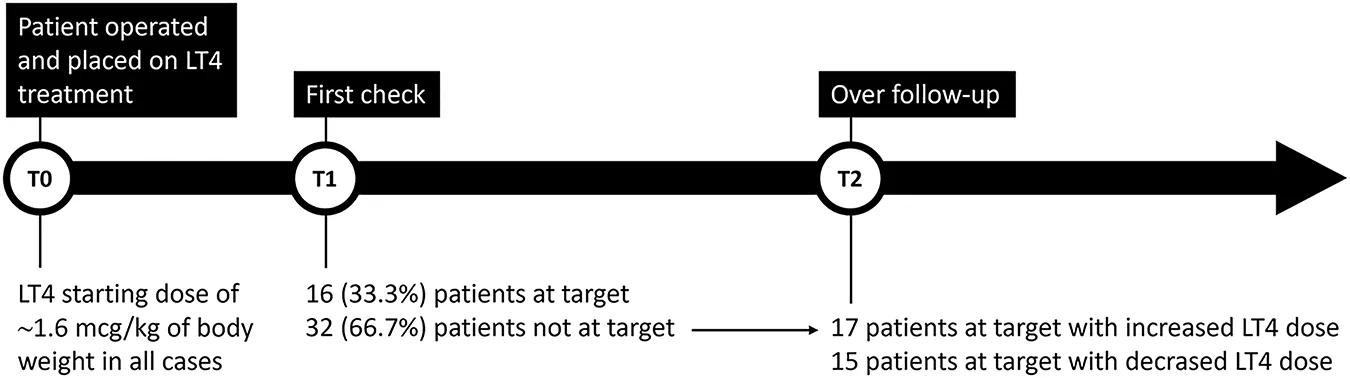
Major study results. T0, thyroidectomy. T1, six weeks after operation. T2, all patients reach the target TSH

When all patients reached the therapeutic target, median TSH, free-T3, and free-T4 were 0.894 mIU/L (0.233 to 1.563), 4.3 pmol/L (3.6 to 4.5), and 20.2 pmol/L (17.7 to 22.1), respectively. The overall median TtT was 4 months (1.5–8); there were 15 patients at target within 6 months, 13 cases within 6–12 months, and other 4 cases needing longer time up to 37 months. The median LT4 dose was 103.6 mcg per day (88–127.7) or 1.57 mcg/kg per day (1.43–1.69) with no significant difference compared to the starting values (p = 0.703 and p = 0.701, respectively). The 32 patients who did not achieved the target TSH at their first follow-up visit included 15 patients showing a decreased LT4 daily requirement (subgroup DECR) and 17 patients with an increased LT4 daily need (subgroup INCR). Figure 1 synthesizes the results observed over follow-up.

### Association of LT4 dose at target and anthropometric data 

**Fig. 2:**
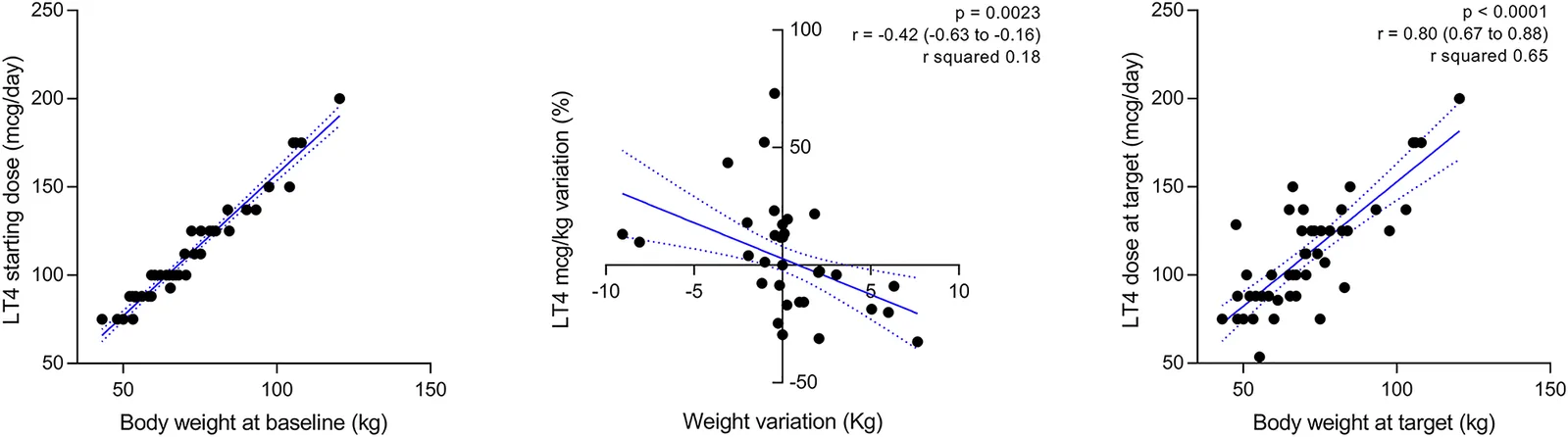
Correlation between LT4 dose and weight. Left: data at baseline. Centre: data according to variations over time. Right: data at target. The continuous blue lines indicate the linear regression and the dotted lines the 95% CI. Linear regression of baseline data is not represented since all patients started with the same LT4 dose

**Fig. 3:**
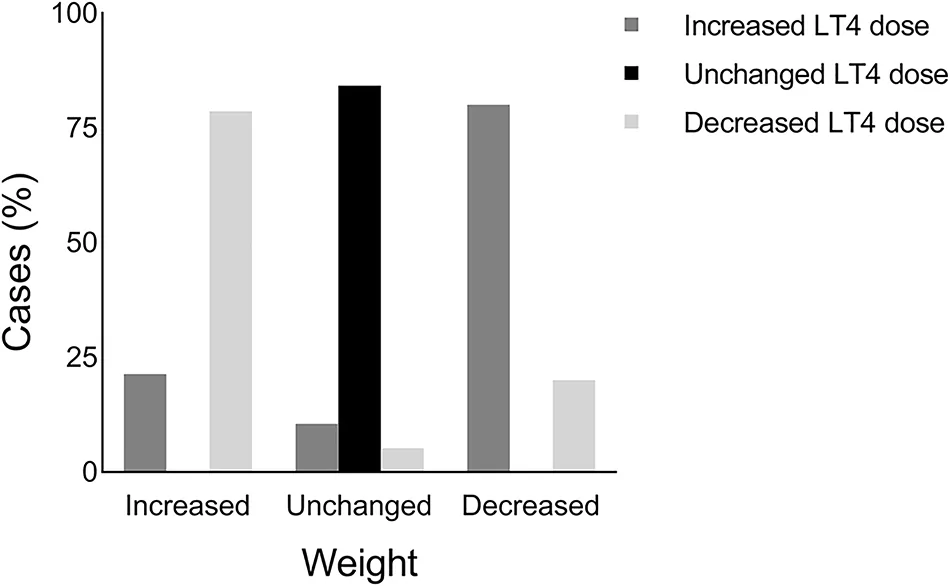
Distribution of increased, unchanged, and decreased LT4 dose according to weight variation. The subgroup of patients with unchanged weight includes cases with a variation in body weight inferior to 0.1 kg compared to the baseline value. LT4 dose is intended as per patient’s body weight

**Fig. 4:**
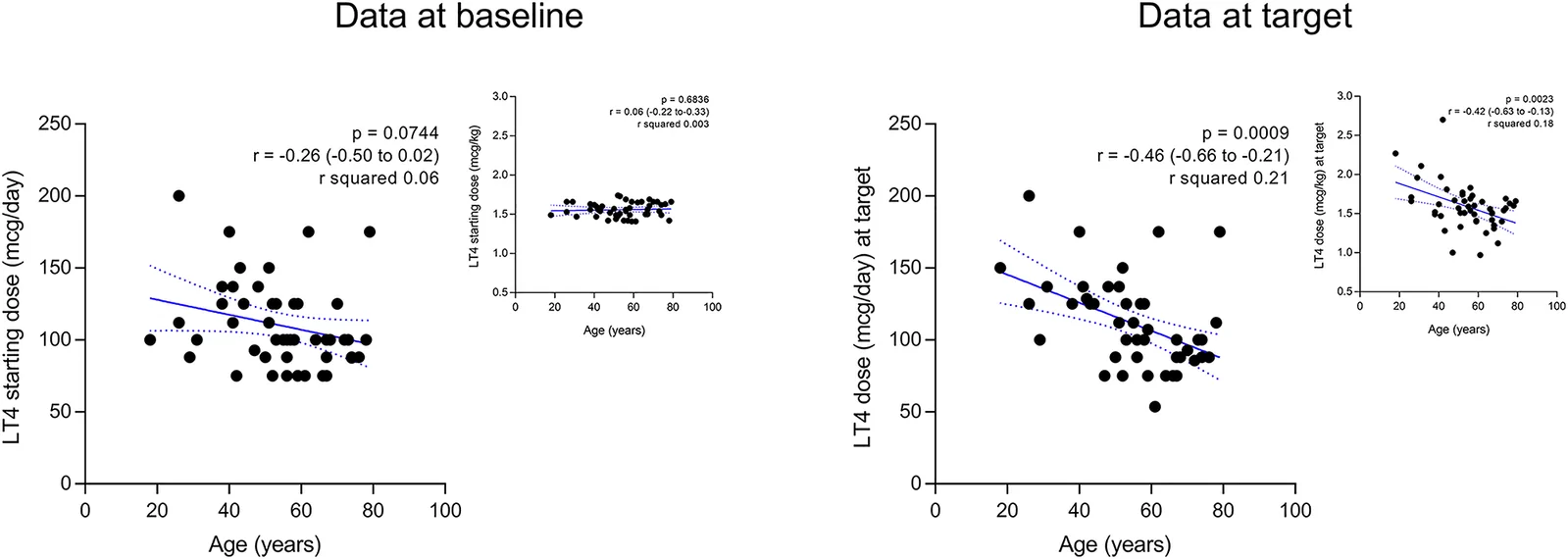
Correlation between LT4 dose and patient age at baseline and at target. The continuous blues lines indicate the linear regression and the dotted lines the 95% CI

No significant difference (p = 0.781) was observed between final body weight (68.12 [56.61–81.06] kg) and the baseline one (67.11 [56.61–78.87] kg); however, 16 patients had body weight increased up to 7.6 kg and other 16 had weight reduction up to 9.1 kg. In these 32 patients the LT4 starting had to be revised. The correlation analysis between body weight and LT4 dose revealed a) a significant association between the daily LT4 dose on target, r = 0.80, p < 0.0001, 95% CI (0.67, 0.88), R² 0.65 and b) significant negative association between LT4 dose variation and weight variation over follow-up, r = –0.429, p = 0.0023, 95% CI (–0.63, –0.16), R² 0.18. Figure 2 shows the results of correlation between LT4 dose and body weight over follow-up. Figure 3 illustrates the distribution of cases at target according to weight variation and consequent LT4 change (p < 0.0001). The correlation analysis between patient age and LT4 dose at target showed a significant negative association considering LT4 daily dose (r = –0.46, p = 0.0009, 95% CI [–0.66, –0.21], R² 0.21) and the dose in mcg per weight (r = –0.42, p = 0.0023, 95% CI [–0.63, –0.13], R² 0.18). Figure 4 shows the findings of correlation analysis between age and LT4 dose at baseline and at target.

### Analysis of subgroups

The INCR subgroup had a significantly higher LT4 dose with respect to the DECR subgroup (p < 0.0001) and the subgroup of patients initially at target (p = 0.0007), with significant difference also between the latter subgroups (p = 0.0002). No significant difference among the three subgroups was found in age, medications interfering with LT4, disorders impairing LT4 efficacy, LT4 preparation, or baseline body weight. The INCR subgroup had significant decrease in body weight while the DECR subgroup had significant increase in body weight. Table 2 illustrates the differences in LT4 dose and body weight observed over follow-up in INCR and DECR subgroups.

**Table 2:** Data of subgroups of patients initially not at target

	Patients with finally increased LT4 dose (n = 17)		Patients with finally decreased LT4 dose (n = 15)	
TSH at target, mIU/L (median, IQR)	0.56, 0.16–2.22		0.36, 0.86–1.57	
Variation of LT4 daily dose (median %, IQR)	Increase of 13.42%, 10.82–22.48		Decrease of 15.66%, 24.70–7.64	
LT4 dose, mcg/kg (median, IQR)	**T0** 1.50, 1.47–1.57	P < 0.0001	**T0** 1.62, 1.54–1.66	P < 0.0001
	**At target** 1.73, 1.60–1.97		**At target** 1.35, 1.25–1.49	
Weight, kg (median, IQR)	**T0** 70.42, 56.04–76.65	P = 0.011	**T0** 65.35, 60.24–80.13	P = 0.001
	**At target** 69.54, 55.10–73.64		**At target** 75, 61.21–82.95	

*IQR*, interquartile range. *T0*, data at thyroidectomy

### Multivariate regression model

Multivariate models were analyzed considering LT4 dose at target as dependent variable. The first multiple linear regression was conducted to examine the combined effect of body weight and age on LT4 dose at target. The model was significant (R² = 0.72, p < 0.0001). Body weight was positively associated with LT4 dose (coefficient = 1.28, p < 0.0001), while age showed a negative association (coefficient = –0.59, p = 0.0009). The second multivariate model included body weight, age, medications with potential to interfere with LT4, and disorders impairing LT4 efficacy. The model was significant (R² = 0.77, p < 0.0001). Body weight was positively associated with LT4 dose (coefficient = 1.33, p < 0.0001), age showed a negative association (coefficient = –0.74, p = 0.0001), comorbidities had positive association (coefficient = 13.8, p = 0.014), medications were not significant. The third model included also LT4 preparation. The results were comparable to the second model with R² = 0.77 and p < 0.0001. Body weight was positively associated with LT4 dose (coefficient = 1.31, p < 0.0001), age showed a negative association (coefficient = –0.73, p = 0.0001), comorbidities had positive association (coefficient = 13.8, p = 0.014), medications and LT4 preparations were not significant.

## Discussion

Hypothyroidism is a common non-communicable disease, usually managed by endocrinologists even if general practitioners represent other stakeholders. Despite guideline recommendations and multiple published strategies for initiating LT4 are available, none allow to reliably achieve euthyroidism in all patients after total thyroidectomy. Evidence from comparative studies further indicates that all approaches yield only modest and broadly similar efficacy [11]. This limitation not only determines inconveniences for patients but also increases healthcare costs due to repeated laboratory testing, clinical visits, and dose adjustments [3, 14]. This study investigated the guideline-recommended weight-adjusted LT4 starting dose in a uniform cohort of post-thyroidectomy patients. Although retrospective, the standardized institutional protocol—initiating LT4 at ~1.6 μg/kg immediately after surgery—reduces allocation bias and strengthens the validity of the findings compared to prior series lacking predefined dosing strategies [11]. Moreover, comprehensive clinical data allowed a thorough evaluation of potential determinants of LT4 effectiveness.

**Fig. 5:**
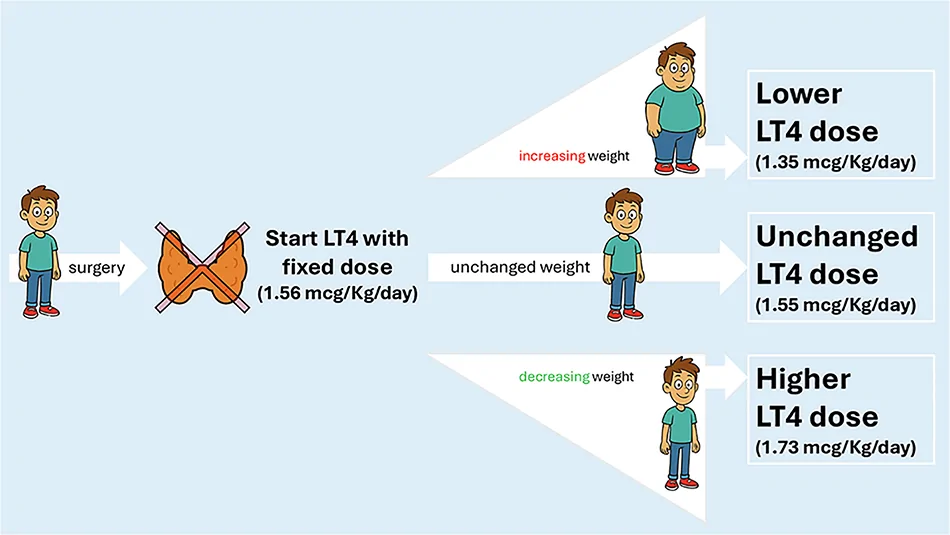
Graphical abstract. This figure synthesizes the major study results.

Our results can be summarized as follows: (1) only one-third of patients reached euthyroidism at first follow-up with the recommended LT4 starting dose; (2) the median TtT was four months, with some cases requiring up to three years; (3) two-thirds of patients necessitated LT4 adjustments due to early postoperative weight changes; (4) body weight and age were significant predictors of LT4 requirements, with body weight being the dominant determinant, and further effect furnished by comorbidities; and (5) concomitant medications, and LT4 formulation showed no significant impact. A graphical abstract is illustrated in Fig. 5.

These findings have several clinical implications. First, the low proportion of patients achieving target TSH at first follow-up corroborates the results from a recent meta-analysis [11], emphasizing the need for clinicians to consider the possible early treatment failure, to inform patients accordingly, and to plan appropriate follow-up. Second, early postoperative weight variations substantially impair the accuracy of weight-based dosing after surgery. Even minimal, weight changes likely reflect differences in lean body mass [15], highlighting the recognized limitation of using body weight alone as a surrogate for LT4 requirements. From a translational perspective, patients not on target after their first postoperative check may have subtle alterations in peripheral thyroid hormone metabolism or responsiveness to LT4 that cannot be predicted preoperatively [16**–**18]. To note, most patients with unchanged weight showed no variation in the replacement dose during the follow-up. In patients experiencing an increased weight following thyroidectomy, the LT4 requirement was lower while the trend was opposite in the fraction of patients with a weight reduction during the follow up. The correlation analyses indicate that 65% of LT4 dose variance can be explained by final body weight and 21% by age. Multivariate regression analysis confirmed both as independent predictors, with body weight as the primary determinant. Together, these factors accounted for ~40% of needs variability, underscoring their clinical relevance but also pointing that other, unmeasured factors may exist. Overall, LT4 requirements show a direct linear relationship with body weight—subject to postoperative change—and an inverse association with age. Since postoperative body weight changes occur in a substantial proportion of patients and are not attributable to LT4 ineffectiveness [15], future studies should investigate the reasons for the different variations in body weight after thyroidectomy. Comorbidities with potential to affect LT4 efficacy can also hold a complementary role. Furthermore, our study demonstrated a high variability even in the time necessary to reach target TSH after thyroidectomy; it may be speculated that a different lag time in reaching pharmacological thyroid homeostasis after thyroidectomy might mirror a different adaptation of peripheral deiodinases activity to the lack of triiodothyronine thyroidal contribution. Until new evidence becomes available, guidelines should underline the need for systematic follow-up of patients after total thyroidectomy, ideally under endocrinology supervision, given that two-thirds of athyreotic patients will vary weight and then require early dose adjustments. Patients should be aware of these data and then prompted to monitor their weight.

This study has limitations. Its retrospective design precludes causal inference, though the standardized protocol applied to all patients minimizes allocation and selection bias. The sample size was smaller than in some prior studies, but rigorous selection ensured a homogeneous cohort with complete data. In addition, our series included a rather high proportion of patients treated with soft-gel LT4, reflecting regional prescribing practices following its introduction in Switzerland. This has likely reduced potential impact of comorbidities and/or concomitant drugs in determining the adequate LT4 dose. Finally, the variation of weight was sometime minimal and then difficult to perceive clinically.

In conclusion, only one-third of patients achieve euthyroidism with the current guideline-recommended LT4 starting dose (1.6 mcg/kg/day) after thyroidectomy. Median time to reach target TSH was four months, with many cases requiring longer. Early postoperative weight changes were frequent and influenced significantly dose requirements, while age provided additional predictive value, and comorbidities may hold a role in this context. These findings highlight the need for early follow-up and rapid LT4 titration in patients who are not at target after initial evaluation. Future prospective studies are warranted to refine dosing strategies and minimize the time required to achieve euthyroidism.

## Data Availability

The datasets generated during and/or analyzed during the current study are not publicly available but are available from the corresponding author on reasonable request.

## References

[1] A. Crafa, A.E. Calogero, R. Cannarella, L.M. Mongioi’, R.A. Condorelli, E.A. Greco, A. Aversa, S. La Vignera, The burden of hormonal disorders: A worldwide overview with a particular look in Italy. Front Endocrinol (Lausanne) **12**, 694325 (2021). 10.3389/fendo.2021.694325 PMC8242938 34220719

[2] L.H. Duntas, J. Jonklaas, Levothyroxine dose adjustment to optimise therapy throughout a patient’s lifetime. Adv Ther **36**(Suppl 2), 30–46 (2019). 10.1007/s12325-019-01078-2 PMC6822824 31485977

[3] B. Biondi, L. Wartofsky, Treatment with thyroid hormone. Endocr Rev **35**(3), 433–512 (2014). 10.1210/er.2013-1083 24433025

[4] J. Jonklaas, A.C. Bianco, A.J. Bauer, K.D. Burman, A.R. Cappola, F.S. Celi, D.S. Cooper, B.W. Kim, R.P. Peeters, M.S. Rosenthal, A.M. Sawka; American Thyroid Association Task Force on Thyroid Hormone Replacement, Guidelines for the treatment of hypothyroidism: prepared by the american thyroid association task force on thyroid hormone replacement. Thyroid **24**(12), 1670–1751 (2014). 10.1089/thy.2014.0028 PMC4267409 25266247

[5] Centanni M., Duntas L., Feldt-Rasmussen U., Koehrle J., Peeters R. P., Razvi S., Trimboli P., Virili C. ETA guidelines for the use of levothyroxine sodium preparations in monotherapy to optimize the treatment of hypothyroidism. Eur Thyroid J. 2025:ETJ-25-0123. 10.1530/ETJ-25-0123. PMC12323320 40622204

[6] O. Olubowale, D.R. Chadwick, Optimization of thyroxine replacement therapy after total or near-total thyroidectomy for benign thyroid disease. Br J Surg **93**(1), 57–60 (2006). 10.1002/bjs.5157 16323163

[7] R.L. Rosenbaum, U.S. Barzel, Levothyroxine replacement dose for primary hypothyroidism decreases with age. Ann Intern Med **96**(1), 53–55 (1982). 10.7326/0003-4819-96-1-53 7053703

[8] D. Mistry, S. Atkin, H. Atkinson, S. Gunasekaran, D. Sylvester, A.S. Rigby, R.J. England, Predicting thyroxine requirements following total thyroidectomy. Clin Endocrinol (Oxf) **74**(3), 384–387 (2011). 10.1111/j.1365-2265.2010.03940.x 21198738

[9] N.A. Zaborek, A. Cheng, J.R. Imbus, K.L. Long, S.C. Pitt, R.S. Sippel, D.F. Schneider, The optimal dosing scheme for levothyroxine after thyroidectomy: A comprehensive comparison and evaluation. Surgery **165**(1), 92–98 (2019). 10.1016/j.surg.2018.04.097. Epub 2018 Nov 6. Erratum in: Surgery. 2022 Jul;172(1):477. doi: 10.1016/j.surg.2022.04.014. PMID: 30413325; PMCID: PMC6289766. PMC6289766 30413325

[10] V. Di Donna, M.G. Santoro, C. de Waure, M.P. Ricciato, R.M. Paragliola, A. Pontecorvi, S.M. Corsello, A new strategy to estimate levothyroxine requirement after total thyroidectomy for benign thyroid disease. Thyroid **24**(12), 1759–1764 (2014). 10.1089/thy.2014.0111 25268754

[11] Chiardi I., Croce L., Caccavale P., Bertini J., Coperchini F., Magri F., Cappelli C., Trimboli P., Rotondi M. Establishing the adequate levothyroxine dose after total thyroidectomy: A systematic review with meta-analysis. J Clin Endocrinol Metab. 2025:dgaf417. 10.1210/clinem/dgaf417. PMC12527440 40682434

[12] C. Virili, P. Trimboli, M. Centanni, Novel thyroxine formulations: a further step toward precision medicine. Endocrine **66**(1), 87–94 (2019). 10.1007/s12020-019-02049-x 31617168

[13] E. von Elm, D.G. Altman, M. Egger, S.J. Pocock, P.C. Gøtzsche, J.P. Vandenbroucke; STROBE Initiative, The Strengthening the Reporting of Observational Studies in Epidemiology (STROBE) statement: guidelines for reporting observational studies. Lancet **370**(9596), 1453–1457 (2007). 10.1016/S0140-6736(07)61602-X 18064739

[14] U. Feldt-Rasmussen, G. Effraimidis, S. Bliddal, M. Klose, Consequences of undertreatment of hypothyroidism. Endocrine **84**(2), 301–308 (2024). 10.1007/s12020-023-03460-1 PMC11076368 37556077

[15] M. Rotondi, L. Croce, C. Pallavicini, L.L. Manna, S.M. Accornero, R. Fonte, F. Magri, L. Chiovato, Body weight changes in a large cohort of patients subjected to thyroidectomy for a wide spectrum of thyroid diseases. Endocr Pract **20**(11), 1151–1158 (2014). 10.4158/EP14125.OR 24936565

[16] T. Piticchio, F. Savarino, S. Volpe, A. Prinzi, G. Costanzo, E. Gamarra, F. Frasca, P. Trimboli, Inflammatory profile assessment in a highly selected athyreotic population undergoing controlled and standardized hypothyroidism. Biomedicines **12**(1), 239 (2024). 10.3390/biomedicines12010239 PMC10813236 38275410

[17] J. Jonklaas, Sex and age differences in levothyroxine dosage requirement. Endocr Pract **16**(1), 71–79 (2010). 10.4158/EP09257.OR 19833578

[18] J. Jonklaas, S. Razvi, Reference intervals in the diagnosis of thyroid dysfunction: treating patients not numbers. Lancet Diabetes Endocrinol **7**(6), 473–483 (2019). 10.1016/S2213-8587(18)30371-1 30797750

